# Genetic evidence for the mating system and reproductive success of black sea bream (*Acanthopagrus schlegelii*)

**DOI:** 10.1002/ece3.6215

**Published:** 2020-04-03

**Authors:** Xi Wang, Su Liu, Yuqing Yang, Lina Wu, Wenhua Huang, Renxie Wu, Guangli Li, Haifa Zhang, Zining Meng

**Affiliations:** ^1^ State Key Laboratory of Biocontrol Institute of Aquatic Economic Animals and the Guangdong Province Key Laboratory for Aquatic Economic Animals School of Life Sciences Sun Yat‐Sen University Guangzhou China; ^2^ Marine Fisheries Development Center of Guangdong Province Huizhou China; ^3^ College of Fisheries Guangdong Ocean University Zhanjiang China; ^4^ Southern Laboratory of Ocean Science and Engineering Zhuhai China

**Keywords:** *Acanthopagrus schlegelii*, Bateman gradient, multiple matings, parentage analysis, polygynandry

## Abstract

Understanding the mating system and reproductive success of a species provides evidence for sexual selection. We examined the mating system and the reproductive success of captive adult black sea bream (*Acanthopagrus schlegelii*), using parentage assignment based on two microsatellites multiplex PCR systems, with 91.5% accuracy in a mixed family (29 sires, 25 dams, and 200 offspring). Based on the parentage result, we found that 93.1% of males and 100% of females participated in reproduction. A total of 79% of males and 92% of females mated with multiple partners (only 1 sire and 1 dam were monogamous), indicating that polygynandry best described the genetic mating system of black sea bream. For males, maximizing the reproductive success by multiple mating was accorded with the sexual selection theory while the material benefits hypothesis may contribute to explain the multiple mating for females. For both sexes, there was a significant correlation between mating success and reproductive success and the variance in reproductive success of males was higher than females. Variation in mating success is the greatest determinant to variation in reproductive success when the relationship is strongly positive. The opportunity for sexual selection of males was twice that of females, as well as the higher slope of the Bateman curve in males suggested that the intensity of intrasexual selection of males was higher than females. Thus, male–male competition would lead to the greater variation of mating success for males, which caused greater variation in reproductive success in males. The effective population number of breeders (*N*_b_) was 33, and the *N*_b_/*N* ratio was 0.61, slightly higher than the general ratio in polygynandrous fish populations which possibly because most individuals mated and had offspring with a low variance. The relatively high *N*_b_ contributes to the maintenance of genetic diversity in farmed black sea bream populations.

## Graphical abstract

Polygynandry was a predominant mating strategy in black sea bream, and a variance in reproductive success was mainly due to multiple mating. For both sexes, a significant correlation was found between the number of mates and the number of offspring.

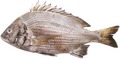

## 1 INTRODUCTION

Mating systems, mainly determined by the frequency of mating in each of the sexes and influenced by environment factors, describe the way in which animal societies are structured in relation to their sex‐specific sexual behavior, mating, and reproductive success (Andersson, 1994; Emlen & Oring, 1977). Monogamy means males and females typically have at most one mate each (some perhaps none at all; little variation in mating success within the sexes). Polygyny occurs males are highly variable in their mating success, whereas females typically have one mate. On the contrary, polyandry occurs when males typically have one mate and females are variable in their mating success. Polygynandry is a mating system which both males and females have multiple partners and they are variable in their mating success. Different mating systems vary in their sexual selection mechanisms (i.e., competition for mating through same‐sex combat and opposite‐sex mate choice), resulting in differences in the intensity and direction of sexual selection (i.e., greater on males or females) which affects the mating and reproductive success of a population (Arnold & Wade, 1984; Jones, 2009). Consequently, mating systems can drive morphological, behavioral, and physiological evolution, and create sexual conflict (Warner et al. 1995; reviewed in Auld, 2018; Karageorge & Wilson, 2017). Many previous studies of mating systems have been based on observations of social behaviors, which can only explain the social mating system and not the genetic mating system, which specifies biological parentage of offspring (Jones & Ardren, 2003; Jones & Avise, 2001). For example, among socially monogamous species, extrapair paternity or maternity discovered with molecular markers and parentage analysis is commonplace (Westneat & Stewart, 2003). Therefore, genetic analysis of parentage is necessary to characterize the genetic mating system of a population and gain insight into the reproductive strategies of individuals that may otherwise be hidden from observation in field studies (Stephen Yezerinac, 2013).

The reproductive success of individuals in a population may be measured by the number of offspring that survive to a particular life stage, such as fingerling in fishes. Reproductive success has a direct relationship with mate choice and mating success, effectively influenced by the mating system and sexual selection (Roney, Oomen, Knutsen, Olsen, & Hutchings, 2018). Since the relationship between mating success and reproductive success is essential for measuring fitness for all sexually studies of sexual selection (Arnold, 1994; Arnold & Duvall, 1994), it presents an important parameter in empirical studies of sexual selection. Moreover, the differences between sexes in the effect of mating success on reproductive success provide a quantitative way of describing the species‐specific mating system (Arnold & Duvall, 1994). Additionally, variance in reproductive success strongly impacts the genetic variation and the effective population size (Haddeland, Junge, Serbezov, & Vã Llestad, 2015). When using mass spawning techniques in a hatchery, the mating systems of a species and the reproductive success of the parents are of concern for selective breeding programs. The greater the genetic variation, the greater the chances of bringing about sustainable improvement through selection. Mating systems with the evenness of mate number and lower variance in reproductive success among multiple mating broodstock increase the effective population size, resulting in a greater genetic variation (Sugg & Chesser, 1994). Whether for elucidating evolutionary mechanisms or for evaluating the maintenance of genetic diversity, it is of great importance to study the mating system and the reproductive success of a species.

Researches of mating systems and reproductive success have been widely carried out in mammals, birds, and snakes (Orians, 1969; Rivas & Burghardt, 2005; Struhsaker & Pope, 1991). However, the mating systems of many fishes remain unknown, including the distribution of reproductive success among adults. Studies on the mating systems of fish by molecular markers have been mainly carried out in Centrarchidae, Gadidae, and Salmonidae. For example, in spotted sunfish (*Lepomis punctatus*), a Centrarchidae fish, the genetic data demonstrated that a great preponderance of the paternity occurred by the nest‐attendant strategy (Male guarding) and that most nests were mothered by multiple dams, which could be considered as polygyny (Dewoody, Fletcher, Mackiewicz, Wilkins, & Avise, 2000). In a Gadidae fish, the Atlantic cod (*Gadus morhua* L.), different matings were recorded with 69 of 70 dams and all of the 30 sires contributing to the offspring; however, 91.2% of the offspring were assigned to a single parental pair, which indicated that the Atlantic cod was polygynandrous and that the variation in reproductive success was very large (Wesmajervi, Westgaard, & Delghandi, 2006).

**FIGURE 1:**
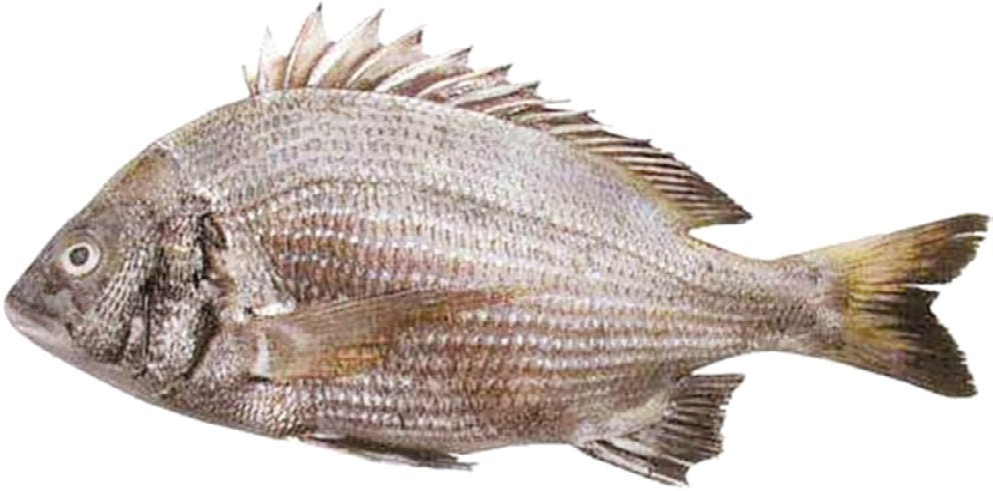
An organism photograph of black sea bream

Among Sparidae fish species (commonly known as sea bream or porgy), various studies have been carried out (Alós, Cabanellas‐Reboredo, & March, 2012; Broadhurst, Butcher, & Millar, 2019; Gardner, Deeming, Wellby, Soulsbury, & Eady, 2015; La Mesa et al., 2011); however, the mating system, variance in reproductive success of males and females, and the fidelity of genetic diversity transmission from parents to offspring remain unknown. For example, black sea bream (*Acanthopagrus schlegelii*) (Figure 1) is an economically important marine fish that is widely distributed in the western Pacific Ocean, ranging from the coasts of Japan and South Korea to the South China Sea. Currently, this species is undergoing inbreeding depression such that selective programs should be assessed to improve the genetic diversity (Law & Sadovy De Mitcheson, 2017). The genetic diversity and fitness of broodstock are in decline, resulting in smaller size, reductions in their market quality and economic value, requiring that measures should be taken to improve genetic health and viability productivity. Therefore, it is necessary to carry out studies on the mating system and reproductive success of black sea bream, which will be helpful to uncover how black sea bream mate and understand the reproductive behavior of Sparidae fish species. Furthermore, based on the information about mating system and reproductive success, breeders can carry on focus management to maximize fertility and improve the genetic diversity (i.e., adjusting the sex ratio in each pond, remaining an adult population size sufficient to achieve a minimum level of *N*_b_).

In this study, we characterize the genetic mating systemof black sea bream and evaluate the possible causes in the variation of reproductive success of black sea bream, by conducting parental assignment using microsatelliteDNA markers. Based on Bateman's principles (Bateman, 1948), we show how the variation in mating success within and between the sexes can affect reproductive success in males and females, and influence the strength and direction of sexual selection. In addition, the effective population size of breeders was calculated to assess the risk for inbreeding in our captive black sea bream population.

## 2 MATERIALS AND METHODS

### 2.1 Broodstock collection and offspring production

The black sea bream broodstock were collected from the South China Sea (Daya Bay, Guangdong) and transferred to the Marine Fisheries Development Center of Guangdong Province in 2017. The fish were fed twice daily according to hatchery operations and cultured in 4 × 4 × 2.5 m^3^ (length × width × depth) concrete‐walled ponds of a recirculating aquaculture systemwith aeration at approximately 19°C in flow‐through seawater under natural photoperiod (14 hr light/10 hr dark) conditions. In January 2018, 29 sires and 25 dams were randomly selected from the broodstock, which were 100% sexually mature. Each breeder was checked by spermiation or oviposition after squeezing the abdomen gently to ensure maturity. All the female black sea breams were artificially induced to estrous by injecting a saline suspension of a hormone solution (2.5 μg LHRH‐A2 and 250 IU HCG per kg body weight). All induced females exhibited mating behavior after synchronization of estrous. According to our observations, black sea breams were clustered and there was no case of mating fish moving away from the group. After 36 hr, the broodstock mated and produced numerous fertilized eggs floating on water, which were all collected by a dense gauze net and then incubated in a 0.5 × 0.5 × 0.5 m^3^ (length × width × depth) aquaria with continuous aeration to ensure sufficient oxygen supply needed for hatching of fertilized eggs at 18–28°C. Under these circumstances, hatching success of the fertilized eggs was about 80%. Feeding was started on the seventh day after hatching and occurred four times a day with a slurry‐type diet made of fish mince and compound feed. In this experiment, the total mortality rate of fry was about 35%. Three months later, 200 of the larvae whose length was about 3 cm were randomly selected for analysis. Remaining offspring were stocked in the Marine Fisheries Development Center for their enhancement and release program.All experiments in the present study were approved by the Animal Care and Use Committee in the Life Sciences School of Sun Yet‐Sen University.

### 2.2 DNA extraction

The pectoral fin of each adult black sea bream was clipped and individually placed in 95% ethanol, and larvae were stored in a bottle under the same conditions. All materials were stored at −20°C for genomic DNA extraction using a DNA extraction kit (Tiangen) following the manufacturer's protocol. The concentrations of the DNA samples were quantified using NanoDrop 2000 spectrophotometer (Thermo Scientific) to ensure their reliability and then diluted to 50 ng/μl for Restriction site Associated DNA Sequencing (RAD‐Seq) (Novogene). DNA from an individual is cut with the chosen restriction enzyme, producing a set of sticky‐ended fragments. To be sequenced on an Illumina machine, these fragments must be ligated to adapters that will bind to an Illumina flow cell. RAD‐Seq uses modified Illumina adapters that enable the binding and amplification of restriction site fragments only. These sheared, sequencer‐ready fragments are then size selected, and this RAD‐Seq library sequenced on the Illumina platform (Davey & Blaxter, 2011). According to the sequencing results, microsatellites were obtained with primers designed by Primer Premier v5.0 (PREMIER Biosoft).

### 2.3 Microsatellite screening and multiplex PCR system development

The total PCR volume for microsatellites screening was 11 μl, including 50 ng DNA template, 0.2 μM primers (synthesized by Tsingke), 5 μl of 2 × Taq PCR StarMix with loading dye (GeneStar), and 3 μl deionized water. The PCR program was as follows: initial denaturation at 94°C for 2 min, followed by 30 cycles at 94°C for 30 s, 55°C for 30 s, 72°C for 45 s, and a final extension at 72°C for 5 min. We used agarose gel electrophoresis to initially eliminate ineffective primers. Next, polyacrylamide gel electrophoresis was used to remove nonspecific fragments. Finally, capillary electrophoresis was applied to select high‐polymorphic microsatellites. After three rounds of screening, the remaining microsatellites were selected to construct multiplex PCR systems. Forward primers for target microsatellites were modified by fluorescent dye (HEX, ROX, FAM). Alleles were detected by GeneMapper v3.2 software (Applied Biosystems).

### 2.4 Genotyping and parentage assignment

The number of alleles (*k*), the observed heterozygosity (*H*_O_), the expected heterozygosity (*H*_E_), the potential deviations from Hardy–Weinberg equilibrium (HWE), the polymorphic information content (PIC), and the nonexclusion probability of each locus were calculated using Cervus v.3.0.3 (Kalinowski, Taper, & Marshall, 2007).Tests for population‐wide linkage disequilibrium between pairs of loci were estimated using GenePop v.4.2.0 (Rousset, 2008). Parentage analysis was carried out using PAPA v.2.0 with default parameter. The parentage allocation method used in PAPA is based on breeding likelihood. Given an offspring genotype, the likelihood of a parental pair of genotypes is defined as the probability of this pair breeding the offspring genotype among all of its possible descents (Duchesne, Godbout, & Bernatchez, 2002).

### 2.5 Analyses of broodstock patterns and sexual selection

The mating and reproductive success of parents were quantified and used to analyze the mating system and estimate sexual selection based on Bateman's principles (Arnold, 1994; Bateman, 1948).Means and variances of mating and reproductive success were calculated for each sex based on the parentage result of 54 broodstock and 200 offspring. When parentage data are available, the sex difference in the opportunity for selection is estimated by calculating the opportunity for selection separately for each sex. The value of the opportunity for selection (*I*_s_) for each sex is expressed as the ratio of the variance in offspring numbers to the squared average in offspring numbers among the members of each sex (variance/mean^2^). The sex difference in the opportunity for sexual selection (*I*_mates_) is expressed as the sex difference in the strength of selection (Δ*I* = *I*_s♂_ − *I*_s♀_) (Shuster, 2009). Sexual selection gradients (Bateman curves) for each sex were determined by simple linear regression of reproductive success against mating success (Gopurenko, Williams, & DeWoody, 2007). We used the parentage assignment results to obtain an estimate of the effective population size of breeders (*N*_b_). The effective number of females, *N*_ef_, was calculated using (Lande & Barrowclough, 1987)

$$N_{\text{ef}}=\left(N_{f}\times k_{f}-1\right)/\left[k_{f}+\left(\frac{V_{f}}{k_{f}}-1\right)\right],$$

where *N*_f_ is the number of sexually mature females, and *k*_f_ and *V*_f_ are the mean and the variance in the number of progenies produced, respectively. The effective number of males, *N*_em_, was calculated analogously.

*N*_b_ was then calculated as (Wright, 1938):

$$N_{b}=4\times \left(N_{\text{ef}}\times N_{\text{em}}\right)/\left(N_{\text{ef}}+N_{\text{em}}\right).$$

## 3 RESULTS

### 3.1 Microsatellite multiplex development

**FIGURE 2:**
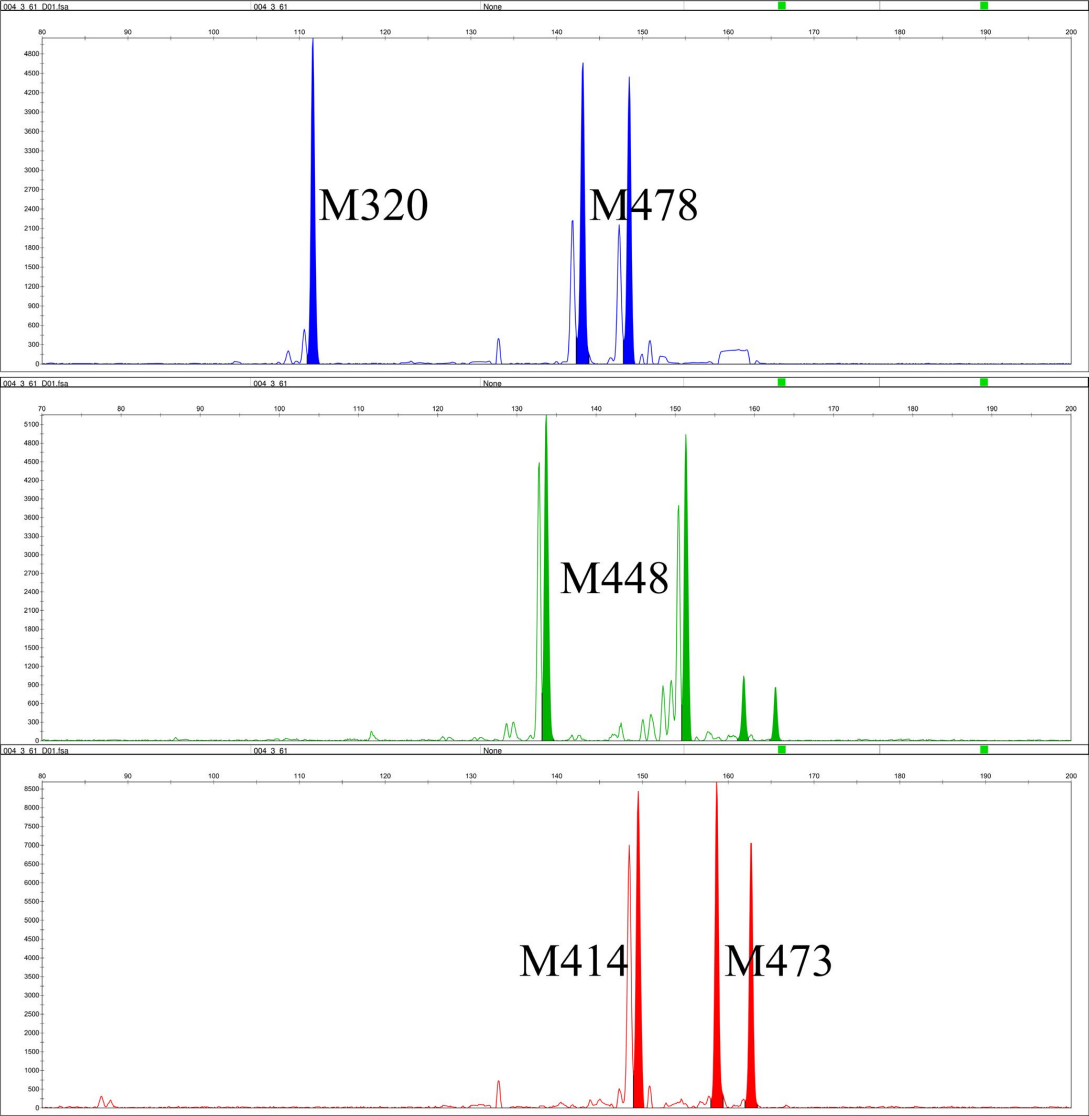
Alleles amplified by the multiplex PCR system A, with five loci in three different colors

**FIGURE 3:**
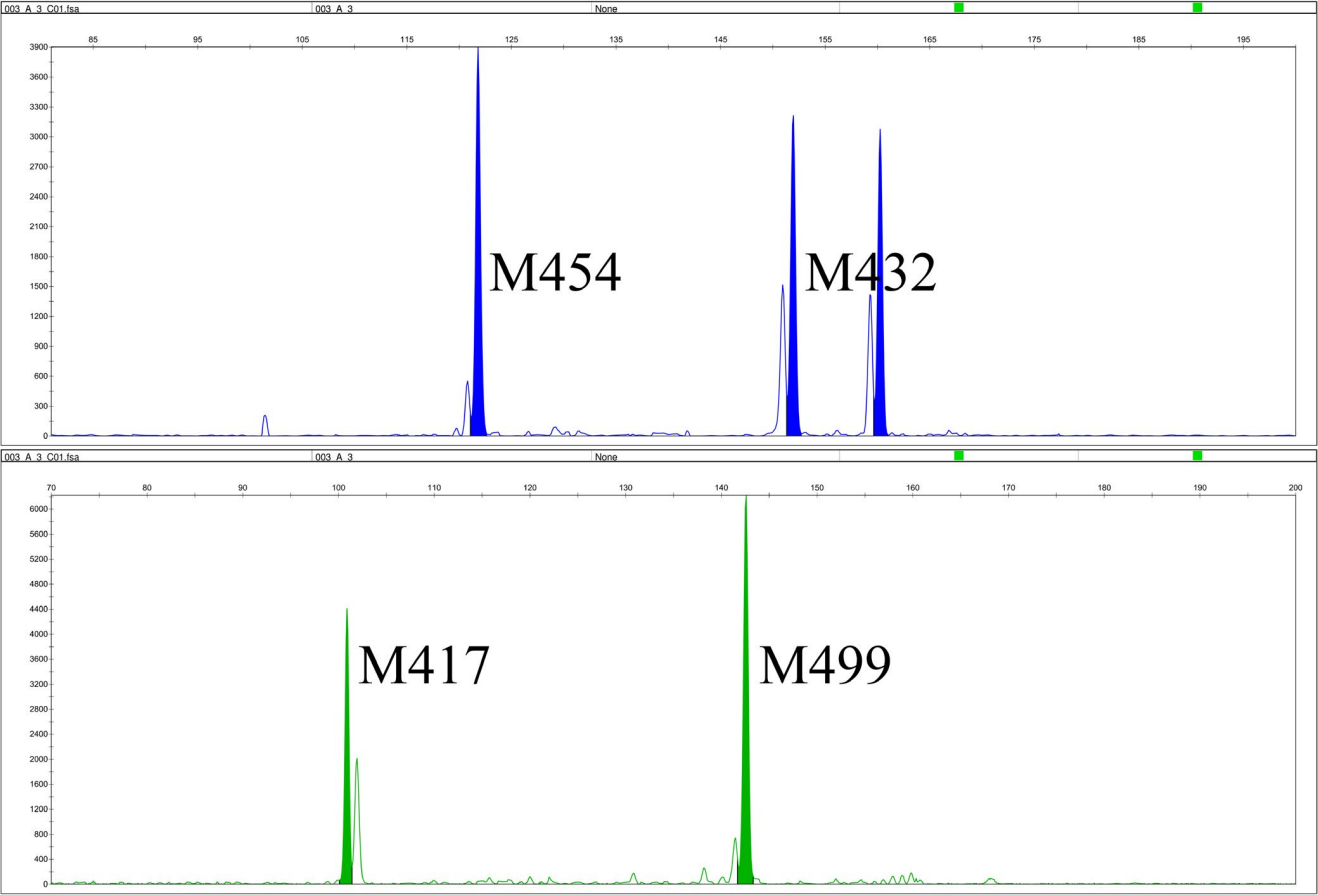
Alleles amplified by the multiplex PCR system B, with four loci in two different colors

A total of 9 microsatellites were selected from RAD‐Seq data to establish two multiplex PCR systems (Table 1). The multiplex PCR reaction A contained the following: 7.5 ng of genomic DNA, 10 μl of 2 × Super Multiplex PCR Mix (CoWin Biosciences), 6.5 μl of deionized water, and 2 μl of primers mix (Table 2) in a final volume of 20 μl. The multiplex PCR reaction B contained the following: 7.5 ng of genomic DNA, 10 μl of 2 × Super Multiplex PCR Mix (CoWin Biosciences), 6.9 μl of deionized water, and 1.6 μl of primers mix (Table 2) in a final volume of 20 μl. The multiplex PCR program was as follows: initial denaturation at 95°C for 10 min, followed by 35 cycles at 95°C for 30 s, 52°C for 30 s, 72°C for 1 min and final extension at 72°C for 5 min. All microsatellite loci were consistently amplified in both PCR systems and all alleles were analyzed on ABI 3730XL Genetic Analyzer (Applied Biosystems) with GeneScan LIZ 500 as size standard. The individual genotype was analyzed by GeneMarker v.2.2.0 software (Hulce, Li, Snyder‐Leiby, & Johathan Liu, 2011) (Figures 2 and 3).

**TABLE 1:** Repeat motif, primers sequences, and annealing temperature (Tm) of microsatellite loci

Locus	Repeat motif	Primer sequences (5′ to 3′)	Dye	Tm/°C	GenBank accession No.
*Multiplex PCR system A*					
M320	(CTC)_4_	F: GCGCTCTCATATTTCAGTTGTCT R: AGATGATTAGCGGTCCAAAGAGT	FAM	56	MH782241
M414	(TCTA)_6_	F: CCCTTTAAATCCAAAATGTCTCC R: AGACTTTAGAGCAACACCATTGC	ROX	54.2	MH782242
M448	(GATA)_7_	F: CGCAAACATCATGAAACATCTTA R: CCTCTAACACCGTTAATTTCCCT	HEX	52.4	MH782245
M473	(TGTC)_7_	F: CTGTTGACCACCTTTTTGTTTTC R: ATTCTCACAGGAGCATCAACATC	ROX	54.2	MH782247
M478	(AAAC)_4_	F: TCCACTGGAGACAGAATGTTTTT R: CTTCGTTTGTTCATCAGTTTTCC	FAM	54.2	MH782248
*Multiplex PCR system B*					
M417	(TTTC)_4_	F: AAACACACAACTTCCTGTCTCCT R: CGTGGATGTGTGTTCCTTTATTT	HEX	56	MH782243
M432	(AAAG)_8_	F: CGTGGATGTGTGTTCCTTTATTT R: AGGCCACAAAAATACCTTCAAAT	FAM	54.2	MH782244
M454	(GAGG)_6_	F: AAAGGGACGTCTACCCTGATG R: AGATGAAAATTGTCAGCGTGTTT	FAM	57.6	MH782246
M499	(TAAA)_8_	F: ATGCAAATTGTTGACAGACATGA R: ATCGGAAGATGATCCACATACAC	HEX	52.4	MH782249

**TABLE 2:** The multiplex PCR system A and B

Multiplex PCR system A		Multiplex PCR system B	
Reagent	Volume (μl)	Reagent	Volume (μl)
2 × Super Multiplex PCR	10	2 × Super Multiplex PCR	10
Primer mix,10 μM each		Primer mix, 10 μM each	
M320	0.2	M417	0.4
M414	0.4	M432	0.4
M448	0.4	M454	0.4
M473	0.4	M499	0.4
M478	0.6	Genomic DNA	1.5
Genomic DNA	1.5	Deionized Water	6.9
Deionized water	6.5		

### 3.2 Genetic characterization of microsatellites and parentage assignment

All samples were genotyped using the above PCR systems. The number of alleles per locus (*k*) ranged from 4 to 15, with an average of 7.78. The observed heterozygosity (*H*_O_) averaged 0.58 and the expected heterozygosity (*H*_E_) averaged 0.70. Four loci showed Hardy–Weinberg disequilibrium due to excess of homozygosity. No evidence of linkage disequilibrium was observed. A total of 8 loci were found with a high level of polymorphic information content (PIC) (Botstein, White, Skolnick, & Davis, 1980) at an average of 0.66. The probability of nonexclusion for each locus ranged from 0.404 to 0.947 when both parents were unknown (NE‐1P), 0.252 to 0.828 when one parent was known (NE‐2P), and 0.094 to 0.704 when both parents were known (NE‐PP) (Table 3). Parentage analysis successfully assigned 91.5% of offspring to a parent pair in the mixed family, and there were only 17 out of 200 offspring could not be matched to their parental pairs.

**TABLE 3:** Characteristics of microsatellite loci in 54 black seam bream individuals

Locus	*k*	*H*_O_	*H*_E_	PIC	HWE	NE‐1P	NE‐2P	NE‐PP
M448	15	0.870	0.880	0.860	NS	0.404	0.252	0.094
M432	9	0.852	0.824	0.793	NS	0.531	0.357	0.176
M499	9	0.315	0.856	0.830	*	0.468	0.302	0.133
M478	10	0.759	0.819	0.787	NS	0.544	0.369	0.188
M414	5	0.759	0.661	0.595	NS	0.761	0.602	0.426
M454	7	0.370	0.682	0.636	*	0.725	0.548	0.352
M417	4	0.481	0.660	0.595	*	0.771	0.611	0.444
M473	7	0.611	0.578	0.539	NS	0.813	0.641	0.450
M320	4	0.167	0.324	0.301	*	0.947	0.828	0.704

Note

NS, not significant (*p* > .05), *significant.

Abbreviations: *H*_E_, expected heterozygosity; *H*_O_, observed heterozygosity; HWE, deviation from Hardy–Weinberg equilibrium; *k*, numbers of alleles; NE‐1P, nonexclusion probability when both parents were unknown; NE‐2P, nonexclusion probability when one parent was known; NE‐PP, nonexclusion probability when both parents were known; PIC, polymorphism information content.

### 3.3 Estimations of mating mechanisms and reproductive success

**FIGURE 4:**
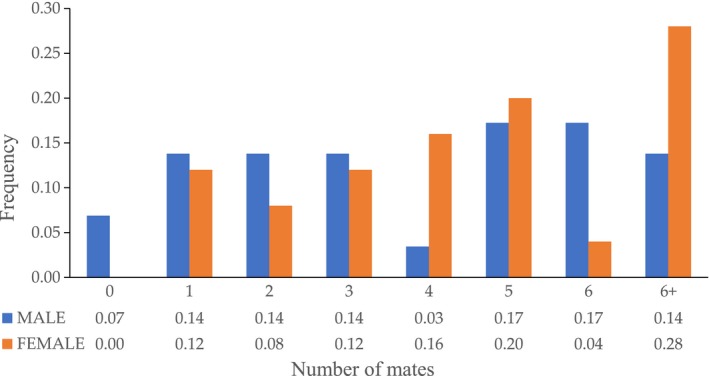
Distribution of mating success. Total frequency of broodstock of each sex mated with 1–6 + mates, 6 + means mated with more than 6 mates

**FIGURE 5:**
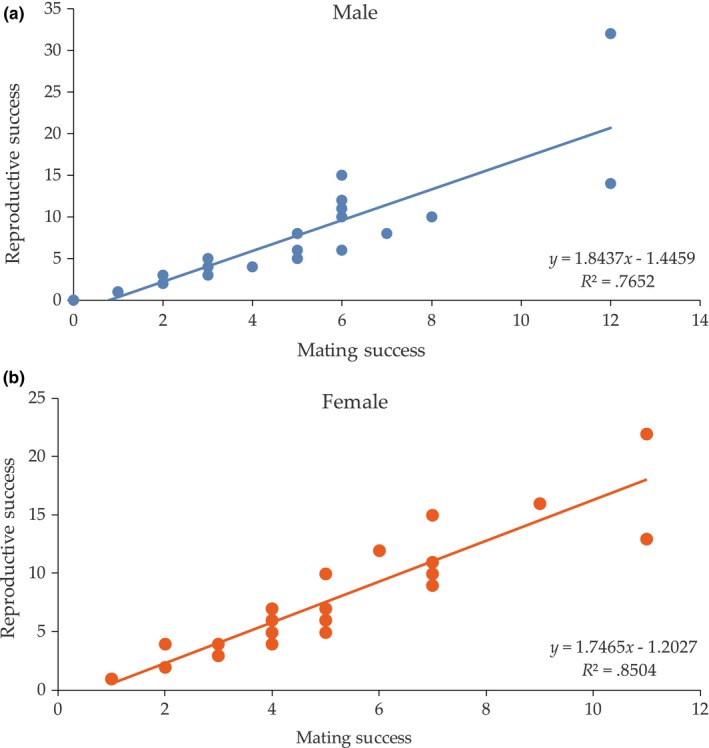
Bateman's gradients for male (a) and female (b) adults. Gradient equals the regression slope of reproductive success (number of offspring) fitted to mating success (number of mates)

The parentage assignments of the mixed family offered genetic evidence to estimate the mating mechanisms and reproductive success. According to the results (Table S1), 93.1% of males (two males had no mates) and 100% of females participated in reproduction. Among them, the most active breeders were female No. 8, which mated with 11 males and produced 22 offspring, and male No. 4, which contributed to 12 families with 32 offspring. We plotted the distribution of mating success for males and females and the frequency of multiple mating among females (92%) was greater than in males (79%). The mating frequency of males increased till five and six partners and then decreased while the frequency of females showed an upward trend (Figure 4). After a simple calculation (Table 4), the results showed that the successfully mated males had, on average, 4.2 ± 3.1 (Standard Deviation, *SD*) partners with an average of 6.3 ± 6.5 offspring while successfully mated females had, on average, 4.9 ± 2.8 partners with an average of 7.3 ± 5.3 offspring. Females mated more partners than males on average but the variation of males is larger, mainly because two males had no mates. The opportunity for sexual selection of males (*I*_s♂_ = 1.05) was twice that of females (*I*_s♀_ = 0.52), and the sex difference in the opportunity for sexual selection (*I*_mates_) was 0.53 (Table 4). Analysis of Bateman's gradients (Figure 5) indicated that the reproductive success was positively correlated with the mating success for both sexes, and the slope of males was slighter higher than females.

**TABLE 4:** Mating and reproductive parameters of 54 black sea bream broodstock

Sex	*N*	*k*_m_	*V*_m_	*SD*_m_	*k*_f_	*V*_f_	*SD*_f_	*I*_s_	*N*_em_	*N*_ef_
Male	29	4.2	9.46	3.1	6.3	42.01	6.5	1.05	15	
Female	25	4.9	7.78	2.8	7.3	27.89	5.3	0.52		18

Abbreviations: *I*_s_, the opportunity for sexual selection; *k*_f_, average offspring amount; *k*_m_, average mates amount; *N*, number of broodstock; *N*_ef_, effective number of females; *N*_em_, effective number of males; *SD*_m_, standard deviation of mates; *SD*_m_, standard deviation of offspring; *V*_m_, variance of mates; *V*_m_, variance of offspring.

For males:

$${\text{RS}}_{m}\;=\;1.8437{\text{MS}}_{m}\;-\;1.4459;\;R^{2}\;=\;0.7652.$$

For females:

$${\text{RS}}_{f}\;=\;1.7465{\text{MS}}_{f}\;-\;1.2027;R^{2}\;=\;0.8054.$$

The effective number of males (*N*_em_) was 15, and the effective number of females (*N*_ef_) was 18 (Table 4). Based on the effective number of males and females, the effective population size of breeders (*N*_b_) was estimated to be 33. The *N*_b_/*N* ratio was 0.61.

## 4 DISCUSSION

### 4.1 Parentage analysis

Our study showed that 91.5% of the progeny in the mixed family were exclusively assigned to their parental pairs. The accuracy obtained in this study was higher than in a previous study of stocked black sea bream in Hiroshima Bay, Japan, in which 69.3% of the offspring were successfully assigned to a single broodstock pair using 7 microsatellite markers which may be caused by the null alleles and allelic dropout resulting from poor quality DNA (Jeong, Gonzalez, Morishima, Arai, & Umino, 2007). Microsatellite genotyping errors occur approximately 2%–3% per locus, and therefore, it was unlikely that determining parentage with 100% accuracy would occur in practice (O'Reilly, Herbinger, & Wright, 1998). Microsatellites have been commonly used for parentage analysis in a range of aquaculture species, while the accuracy has varied significantly across studies (Liu et al., 2012; Pruett et al., 2010; Shao et al., 2017; Yang et al., 2014). In a study of common carp (*Cyprinus carpio* L.), the assignment success rate for 550 offspring from a full factorial cross of 10 dams × 24 sires with 8 microsatellite markers was 95.3% (Vandeputte et al., 2004). A total of 5 microsatellite loci were selected for a parentage assignment test of pacific threadfin (*Polydactylus sexfilis*), and 90% of the offspring from three consecutive spawning events were successfully assigned to their specific parent pairs (Wang, Iwai, Zhao, Lee, & Yang, 2010). Through the differences in the accuracy of parentage assignments above, we considered that, although a great many microsatellite loci can be easily obtained though genomic sequencing, some of them are not suitable for parentage assignment. However, if the discrimination power is more than 80%, the markers for parentage assignment may have high application value in practice (Chen, Cho, & McCouch, 2002), as were the two multiplex PCR systems made up of 9 microsatellite loci used in our study of black sea bream.

### 4.2 Mating system and sexual selection

According to the Bateman curves (Figure 5), the relationship between mating success and reproductive success was linear in both sexes, which accorded with the linear feature of polygynandry (Arnold, 1994). Therefore, we considered polygynandry as the predominant mating strategy of black sea bream in anaquaculture setting. The number of partners differed among sex. Overall, the number of partners for the successful females varied from 1 to 11, while partners for males varied from 0 to 7. The sex difference in the opportunity for sexual selection (*I*_mates_) was 0.53 > 0, indicating a sexual selection modifies males, that is males are the competing sex and females are the choosy sex (Wade, 1979). Hence, there may be male–male competition, leading to the greater variation of mating success for males,which in turn causes greater variation in reproductive success in males and hence slightly greater sexual selection on males.

In males, maximizing reproductive success by multiple mating is in general accordance with Bateman's sexual selection theory. The reproductive success of male was generally limited by fertilizations rather than by sperm production. As a result, males would actively fertilize more females in order to achieve a greater reproductive success. The more females they fertilize, the more genes they may leave behind (Arnold, 1994). Black sea bream is a mass spawning species with no parental care, as a result, the male should seek to mate with as many partners as possible. In mass spawns, the participating males release large amounts of sperm with each male attempting to increase his relative paternity through numeric dilution of rival's sperm. This would in turn limit the total number of matings in which a male can effectively participate (Warner & Harlan, 1982), and it may be a proper explanation for why the mating frequency of males increased till five and six partners and then decreased.

While males are expected to be promiscuous because reproductive success is directly related to the number of mates, the adaptive significance of females copulating with multiple males is less clear (Parker, 1992).There were two hypotheses to explain why females favor multiple mating: the material benefits view and the genetics benefits concept (Reynolds, 1996). The material benefits view is that a female may obtain additional care (nutrients in the seminal fluid, protection and food resources) by mating with more than one male or she may obtain an adequate sperm supply to fertilize of all her eggs. The genetics benefits concept states that when females encounter better males than their previous mates, they should mate again so that their eggs may be fertilized by the better males' sperm (Yasui, 1997). In addition, genetic benefits concept implies that increased offspring diversity resulting from multiple mating enhances female fitness by reducing sibling competition or by serving as a hedge against environmental uncertainty. In reproductive season, the estrus for female sea bream is about 3–5 days while male estrus can last for months, so females are more urgent to reproduce. Besides, unlike mammals or birds, postspawning care is lacking in black sea bream, which means the females do not put effort to raise their offspring, indicating that the reproductive contribution of females is nearly equal to that of males. Therefore, multiple mating may just be important for fertilization assurance for females (material benefits expected). Mass spawning males do not control access by other males to females but rather compete among themselves for the fertilization of the eggs after release (van den Berghe & Warner, 1989), which provides the opportunity for females to simultaneously mate with many males. As a result, the mating frequency of female showed an upward trend when female chose to mate as many partners as possible. When individuals get material benefits through multiple mating, genetic benefits may be gained at the same time. A higher genetic diversity within a progeny array stemming from multiple mating by females might also serve to reduce the potential cost of inbreeding (Garant, Dodson, & Bernatchez, 2001). However, in our current study, the data are inadequate to determine whether or not offspring from one cross have greater survival or fitness than another cross. In future study, we will compare the level of genetic diversity in females with single mating to those with multiple mating to examine the hypothesis in future research. As a conclusion, material benefits hypothesis may contribute to explain the multiple mating for females and genetic benefits concept needs further verification.

According to Bateman's principles, intrasexual selection could occur when reproductive success within a sex was positively correlated with mating success (Bateman, 1948). Therefore, he predicted that the intensity of intrasexual selection could be measured as the rate of increase in reproductive success with mating success (the slope of the Bateman curve) and that the sex under the more intense selection regime will present a higher slope. In our study, the slope of the Bateman curve in males was slightly higher than in females, showing that the intensity of intrasexual selection of males was slightly higher than females, which was consistent with the conclusion as *I*_mates_ got. However, although there may be intrasexual selection, we considered the strength was weak because nearly all broodstock mated (only two males had no mates). As for the factors related to the intrasexual selection, we could not make assumptions based on existing data so we would put efforts on this problem in the following research.

### 4.3 Reproductive success

It was clear that in black sea bream, the individuals that mated with more partners also produced more progeny. When the relationship between mating success and reproductive success is strongly positive, the variation in mating success is the greatest determinant to reproductive success. However, based on the existing experimental data, we can only compare the variance of individual reproduction success of different mating systems (different variation in mating success) but we have no idea to infer the exact traits and mechanisms that affect the variance, which needs further evaluation. In our study, the most successful male produced 16% of the offspring, while the top female contributed 11%. One‐third of the males sired approximately 70% of the offspring and similarly, 32% of the females contributed 60% of the offspring. Although variance in reproductive success was observed, it seemed smaller than other species with different variation in mating success. According to a study with a polygynous fish, Japanese flounder (*Paralichthys olivaceus*), the contribution of the candidate broodstock to the next generation was highly skewed as the contribution to almost all of the offspring was monopolized by a single male, and half of the females did not produce any offspring (Sekino et al., 2003). Nile tilapia (*Oreochromis niloticus*), whose main mating strategy was polyandry, exhibiting a huge variance of reproductive success among females. In a study of *O. niloticus*, 95.45% of offspring bred by one female and one‐third of males siring more than 70% of the offspring (Fessehaye et al., 2006).

We suspected that the size of the aquaculture pond may be related to the reproductive success of breeding black sea bream because during a breeding period, numerous individuals ovulated and ejaculated at a very similar time, and then, the narrow environment forced individuals to crowd together, increasing the chance of mass fertilization, leading to more extensive multiple mating and therefore reduces the variance of reproductive success. However, we have previously observed (unpublished data) that in the orange‐spotted grouper (*Epinephelus coioides*), whose breeding conditions were almost the same as this study, there was a large variance in the reproductive success of individuals because a pair of parents contributed more than 90% offspring. Compared to the changes in the size of breeding space, we considered that mating system had greater impact on the variance in reproductive success.

### 4.4 Effective population size

The effective population size of breeders (*N*_b_) is the size of the ideal population that would undergo the same amount of random genetic drift as the actual population, which depends strongly on mating systems and is used to assess the risk for inbreeding in a population (Lande & Barrowclough, 1987). In general, the *N*_b_ of a polygynandrous population is close to half the number of broodstock (*N*). However, many studies have clearly noted that inequalities in reproductive success may result in a decrease in the effective population size (Nunney, 1993). *N*_b_ (33) was obtained in this study, and the *N*_b_/*N* ratio (0.61) of black sea bream was slightly higher than the general ratio in polygynandrous populations and was also higher than that previously reported for several fish species, such as 0.43–0.45 for Nile tilapia (*Oreochromis niloticus*) (Fessehaye et al., 2006), 0.31–0.42 for large yellow croaker (*Larimichthys crocea*) (Liu, Zhao, Cai, & Wang, 2013), and 0.12 for Senegalese sole (*Solea senegalensis*) (Porta, María Porta, Martínez‐Rodríguez, & Carmen Alvarez, 2006). This may be explained by the low variance in reproductive success among spawning broodstock (most individuals mated and had offspring). Moreover, the relatively high *N*_b_ contributes a low risk for inbreeding in farmed black sea bream populations.

## 5 CONCLUSIONS

This study provides a demonstration of a genetic approach to analyze the mating system and quantify the variance in individual mating and reproductive success of black sea bream. The genetic evidence uncovered that polygynandry appeared to be a predominant mating strategy in black sea bream. In fish, males are expected to be promiscuous because their reproductive success is directly related to their number of mates. Likewise, females may mate with multiple males to obtain an adequate sperm supply which was accorded with the material benefits but the genetics benefits concept needed further evidence. The variation in individual reproductive success of black sea bream is smaller than other species with different mating systems. In future studies, we will focus on evaluating the contribution of genetic benefits concept to females' mating strategy and the relationship between sexual selection and traits, such as body length, weight, or age.

## CONFLICT OF INTEREST

The authors declare no conflicts of interest.

## AUTHOR CONTRIBUTION

**Xi Wang:** Conceptualization (supporting); Data curation (supporting); Formal analysis (supporting); Investigation (supporting); Project administration (supporting); Software (supporting); Validation (supporting); Visualization (supporting); Writing‐original draft (supporting); Writing‐review & editing (supporting). **Su Liu: **Data curation (equal); Methodology (equal); Resources (supporting). **Yuqing Yang:** Data curation (equal); Methodology (equal); Resources (equal). **Lina Wu:** Data curation (equal); Formal analysis (equal); Methodology (equal); Software (equal). **Wenhua Huang:** Data curation (equal); Formal analysis (equal); Methodology (equal). **Renxie Wu:** Investigation (equal); Resources (supporting). **Guangli Li:** Investigation (equal); Resources (equal). **Haifa Zhang:** Investigation (equal); Resources (equal). **Zining Meng:** Conceptualization (lead); Data curation (supporting); Funding acquisition (lead); Investigation (lead); Methodology (lead); Project administration (lead); Resources (lead); Supervision (lead); Visualization (lead); Writing‐original draft (supporting); Writing‐review & editing (supporting).

## Supporting information

Supplementary Material

## Data Availability

DNA sequences: Genbank accessions MH782241–MH782249.
