# Supplementary material for: Genetic evidence for the mating system and reproductive success of black sea bream (Acanthopagrus schlegelii)
Source: Ecol Evol. 2020 Apr 3;10(10):4483–94. doi: 10.1002/ece3.6215 (PMC7246203; doi:10.1002/ece3.6215)
Supplement: Supplementary file 1 — Supplementary Material [file ECE3-10-4483-s001.docx]

**Appendix A**

**Table A1.** Matrix of parental crosses and number of offspring designated to each cross (parent-offspring array).

| Male↓ | Female→ |  |  |  |  |  |  |  |  |  |  |  |  |  |  |  |  |  |  |  |  |  |  |  |  |  |
| --- | --- | --- | --- | --- | --- | --- | --- | --- | --- | --- | --- | --- | --- | --- | --- | --- | --- | --- | --- | --- | --- | --- | --- | --- | --- | --- |
| Name | 1 | 2 | 3 | 4 | 5 | 6 | 7 | 8 | 9 | 10 | 11 | 12 | 13 | 14 | 15 | 16 | 17 | 18 | 19 | 20 | 21 | 22 | 23 | 24 | 25 | Total |
| 1 |  |  |  |  |  |  |  |  |  | 1 |  |  |  |  |  |  |  |  |  |  |  |  |  |  |  | 1 |
| 2 | 1 |  |  |  |  |  |  | 2 | 1 | 1 |  |  |  |  |  |  |  |  |  |  | 3 |  |  |  |  | 8 |
| 3 |  |  |  |  |  |  |  | 1 |  |  |  |  |  |  |  |  |  | 1 |  |  |  |  |  |  |  | 2 |
| 4 | 4 |  | 1 | 1 | 2 |  |  | 7 |  | 4 |  | 1 |  |  |  | 5 |  |  |  | 1 | 2 | 2 |  |  | 2 | 32 |
| 5 |  | 1 |  | 1 | 1 | 1 |  | 1 |  | 1 | 1 | 2 | 1 |  |  | 2 |  |  |  |  | 1 |  |  | 1 |  | 14 |
| 6 | 1 |  | 1 |  |  |  | 1 |  |  | 1 | 1 |  |  |  |  | 2 |  |  |  |  |  | 1 |  |  | 2 | 10 |
| 7 |  |  |  | 1 |  |  |  |  |  |  |  | 1 |  |  |  |  |  | 2 | 1 |  |  |  |  | 1 |  | 6 |
| 8 |  |  |  | 1 |  |  |  | 2 | 1 |  |  |  |  |  |  |  |  |  |  |  |  |  |  |  |  | 4 |
| 9 |  |  |  |  |  |  |  |  |  |  |  |  |  |  |  |  |  |  | 1 |  | 1 |  |  |  |  | 2 |
| 10 |  |  |  |  |  |  |  |  |  |  |  |  |  |  |  |  |  |  | 1 |  |  |  |  |  |  | 1 |
| 11 |  |  |  |  |  |  |  |  |  |  |  |  |  |  |  |  |  |  |  |  |  |  |  |  | 1 | 1 |
| 12 |  | 1 |  | 5 |  | 1 |  |  |  |  |  |  |  |  |  | 1 |  |  | 6 |  |  |  |  |  | 1 | 15 |
| 13 |  |  |  |  |  | 1 |  | 1 |  |  | 3 | 1 |  |  |  | 2 |  |  |  |  |  |  |  |  | 2 | 10 |
| 14 |  |  |  | 1 |  |  |  | 1 |  |  |  | 1 |  |  |  |  | 1 |  | 1 |  |  |  |  |  |  | 5 |
| 15 |  | 1 |  |  | 1 |  | 1 | 1 |  |  |  |  |  |  |  | 1 |  |  |  |  |  |  |  |  | 1 | 6 |
| 16 |  | 1 |  |  |  |  |  |  |  |  | 1 | 1 |  |  |  |  | 1 |  |  |  |  |  |  | 1 |  | 5 |
| 17 |  |  |  |  | 1 | 1 | 1 |  |  |  |  |  |  |  |  |  |  |  |  |  |  |  |  |  |  | 3 |
| 18 | 1 | 1 |  | 5 |  |  |  |  |  | 2 |  | 1 |  |  |  | 1 |  |  |  |  |  |  |  |  |  | 11 |
| 19 |  |  |  |  |  |  |  | 1 |  |  |  |  | 2 |  |  |  |  |  |  |  |  |  |  |  |  | 3 |
| 20 |  |  |  |  |  |  |  |  |  |  |  |  |  |  |  |  | 1 |  |  |  |  | 2 | 1 |  |  | 4 |
| 21 |  |  |  |  |  |  |  |  |  | 1 | 1 | 1 |  |  |  |  |  |  |  |  |  |  |  |  | 1 | 4 |
| 22 |  |  |  |  |  | 1 | 1 |  |  |  |  | 1 |  | 2 |  | 1 |  |  |  |  |  | 1 |  | 1 |  | 8 |
| 23 |  |  |  |  |  |  |  |  |  |  |  |  | 1 |  |  | 1 |  |  |  |  |  |  |  |  |  | 2 |
| 24 |  |  |  |  |  |  |  |  |  |  |  | 2 |  | 2 | 1 |  |  |  |  |  |  |  |  |  |  | 5 |
| 25 |  | 2 |  |  |  |  |  | 2 |  |  |  | 1 | 1 |  |  |  |  |  |  |  | 4 |  |  | 2 |  | 12 |
| 26 |  | 2 | 1 |  | 1 |  |  | 3 |  |  |  |  |  |  |  |  |  |  |  |  | 1 |  |  |  |  | 8 |
| 27 |  |  |  |  |  |  |  |  |  |  |  |  |  |  |  |  |  | 1 |  |  |  |  |  |  |  | 1 |
| 28 |  |  |  |  |  |  |  |  |  |  |  |  |  |  |  |  |  |  |  |  |  |  |  |  |  | 0 |
| 29 |  |  |  |  |  |  |  |  |  |  |  |  |  |  |  |  |  |  |  |  |  |  |  |  |  | 0 |
| Total | 7 | 9 | 3 | 15 | 6 | 5 | 4 | 22 | 2 | 11 | 7 | 13 | 5 | 4 | 1 | 16 | 3 | 4 | 10 | 1 | 12 | 6 | 1 | 6 | 10 | 183 |
